# Artificial Intelligence and Machine Learning in the Diagnosis and Management of Stroke: A Narrative Review of United States Food and Drug Administration-Approved Technologies

**DOI:** 10.3390/jcm12113755

**Published:** 2023-05-30

**Authors:** Anirudha S. Chandrabhatla, Elyse A. Kuo, Jennifer D. Sokolowski, Ryan T. Kellogg, Min Park, Panagiotis Mastorakos

**Affiliations:** 1 School of Medicine, University of Virginia Health Sciences Center, 1215 Lee Street, Charlottesville, VA 22903, USA; 2 Department of Neurological Surgery, University of Virginia Health Sciences Center, 1215 Lee Street, Charlottesville, VA 22903, USA; 3 Department of Neurological Surgery, Thomas Jefferson University Hospital, 111 S 11th Street, Philadelphia, PA 19107, USA

**Keywords:** machine learning, artificial intelligence, stroke, intracerebral hemorrhage, FDA

## Abstract

Stroke is an emergency in which delays in treatment can lead to significant loss of neurological function and be fatal. Technologies that increase the speed and accuracy of stroke diagnosis or assist in post-stroke rehabilitation can improve patient outcomes. No resource exists that comprehensively assesses artificial intelligence/machine learning (AI/ML)-enabled technologies indicated for the management of ischemic and hemorrhagic stroke. We queried a United States Food and Drug Administration (FDA) database, along with PubMed and private company websites, to identify the recent literature assessing the clinical performance of FDA-approved AI/ML-enabled technologies. The FDA has approved 22 AI/ML-enabled technologies that triage brain imaging for more immediate diagnosis or promote post-stroke neurological/functional recovery. Technologies that assist with diagnosis predominantly use convolutional neural networks to identify abnormal brain images (e.g., CT perfusion). These technologies perform comparably to neuroradiologists, improve clinical workflows (e.g., time from scan acquisition to reading), and improve patient outcomes (e.g., days spent in the neurological ICU). Two devices are indicated for post-stroke rehabilitation by leveraging neuromodulation techniques. Multiple FDA-approved technologies exist that can help clinicians better diagnose and manage stroke. This review summarizes the most up-to-date literature regarding the functionality, performance, and utility of these technologies so clinicians can make informed decisions when using them in practice.

## 1. Introduction

Stroke is a neurological emergency and the fifth leading cause of death in the United States [1,2,3]. Established clinical interventions exist for many stroke subtypes, such as large vessel occlusion (LVO) and intracranial hemorrhage (ICH). Prompt treatment is one of the more important factors in maximizing the preservation of neurological function. Notably, each minute of treatment delay results in significant neuronal death and the loss of 4.2 days of healthy life [4].

Tools to improve the speed and accuracy of stroke diagnosis and treatment could improve patient outcomes. Artificial intelligence/machine learning (AI/ML) will play a large role in developing such tools. AI/ML in healthcare is growing at 40% per year, and its adoption has the potential to cut USD 150 billion in healthcare costs by 2026 [5]. Recognizing the potential AI/ML has to improve healthcare, the United States Food and Drug Administration (FDA) has developed new protocols to assess the safety and efficacy of AI/ML-enabled health technologies [6]. AI/ML-enabled algorithms have been leveraged for various clinical applications such as detecting liver fibrosis [7], analyzing EKGs [8], monitoring Parkinson’s [9], diagnosing glaucoma [10], and classifying lung cancer [11]. The FDA has approved 22 AI/ML-enabled technologies for indications specifically related to stroke diagnosis and rehabilitation. Existing literature reviews in this area have broadly evaluated AI/ML algorithms that have largely been developed for research purposes [12,13,14,15,16]. No study to date has comprehensively evaluated the real-world clinical performance of clinically available, FDA-approved devices indicated for the diagnosis and management of stroke. This review aims to synthesize the most relevant, up-to-date information related to these technologies and provide an overview of their unique functionalities and performances regarding improving clinical workflows and outcomes.

## 2. Methods

### 2.1. Technology Search

We sought to identify all FDA-approved, AI/ML-enabled medical technologies with indications for ischemic stroke and/or ICH. To compile this list, our search had two components.

**Figure 1:**
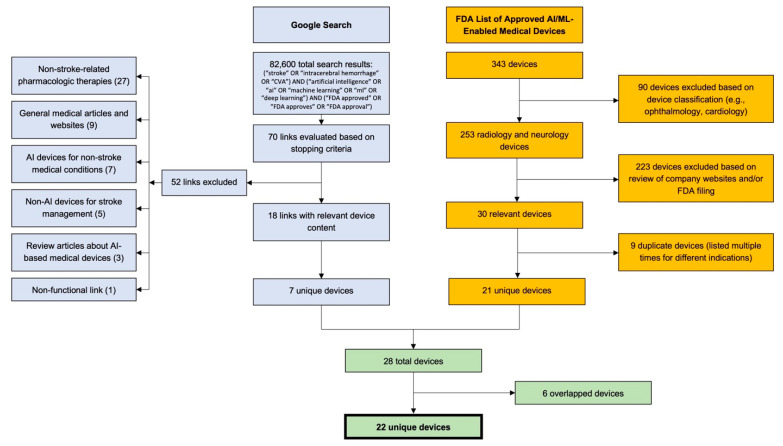
Literature and web search methodologies. A Google search was conducted using a compound search with terms related to stroke, artificial intelligence/machine learning, and FDA approval. Seventy links were evaluated based on stopping criteria, which resulted in the discovery of 7 unique technologies. A total of 343 technologies were evaluated from an FDA database, which resulted in the discovery of 21 unique technologies. The final list of 22 technologies was created after excluding six overlaps from the two searches. CVA: Cerebrovascular Accident.

First, we examined the previously cited [17] database that is directly maintained by the FDA and contains 343 AI/ML-enabled technologies that the FDA has approved. We extracted technologies labeled “radiology” and “neurology” (n = 253). As previously described in the literature, this list does not have search or filter functionality to assess technology descriptions or approval letters [18]. Therefore, two reviewers analyzed the 253 official FDA approval letters and/or company websites to determine their relevance to ischemic stroke/ICH. This search resulted in 30 technologies, 9 of which were listed for multiple indications, resulting in 21 unique technologies (Figure 1).

Second, we conducted an internet search in accordance with previously described methods [19] to identify technologies with approval statuses not covered in the FDA list. In short, we conducted a Google search with the compound search term: (“stroke” OR “intracerebral hemorrhage” OR “CVA”) AND (“artificial intelligence” OR ”ai” OR ”machine learning” OR ”ml” OR ”deep learning”) AND (“FDA approved” OR “FDA approves” OR “FDA approval”) (Figure 1). The search returned 82,600 results, and URLs were sequentially assessed for information regarding AI/ML-enabled, FDA-approved technologies. As per previously described stopping criteria [19], we concluded the search after 40 new results (i.e., 4 full pages of Google results) failed to reveal new technologies. We evaluated 70 links; 18 links included relevant technologies, 7 of which were unique (Figure 1).

Combining the results from both search components, we assessed 28 ischemic stroke-/ICH-related technologies. After excluding 6 duplicates, we arrived at a total of 22 unique technologies (Figure 1).

### 2.2. Literature Search

We queried PubMed and relevant company websites to assess the most up-to-date (post-2018), technology-related literature published in peer-reviewed journals. The PubMed search was conducted by querying the database with the device name (e.g., “Rapid AI”), and company websites were searched for “research” or “data” pages that cited studies involving the company’s technology. Studies published before 2018 were excluded, and we did not include review articles, editorials, or letters to the editor. Original, primary research that directly assessed the clinical performance of an AI-enabled technology for stroke diagnosis or management in humans was included. For each publication, we collected data regarding metrics commonly used to assess ML algorithm performance, such as accuracy, specificity, sensitivity, positive predictive value, negative predictive value, and area under the receiver-operator curve (AUC). We report results from 45 publications. This study was IRB-exempt.

## 3. Review of Literature

Our search revealed 22 FDA-approved, AI/ML-enabled technologies indicated for stroke diagnosis and management. A total of 18 companies developed these 22 technologies, with a majority (11/18; 61%) headquartered outside of the United States. The first approval was in February 2018. All initial approvals were for technologies that assist with ischemic stroke diagnosis, but two out of the last three approvals (BrainQ and IpsiHand) were for devices indicated for post-stroke rehabilitation (Table 1). Here, we synthesize the most recent literature on the clinical performance of these technologies.

## 4. Large Vessel Occlusion (LVO) Identification in Acute Ischemic Stroke

**Figure 2:**
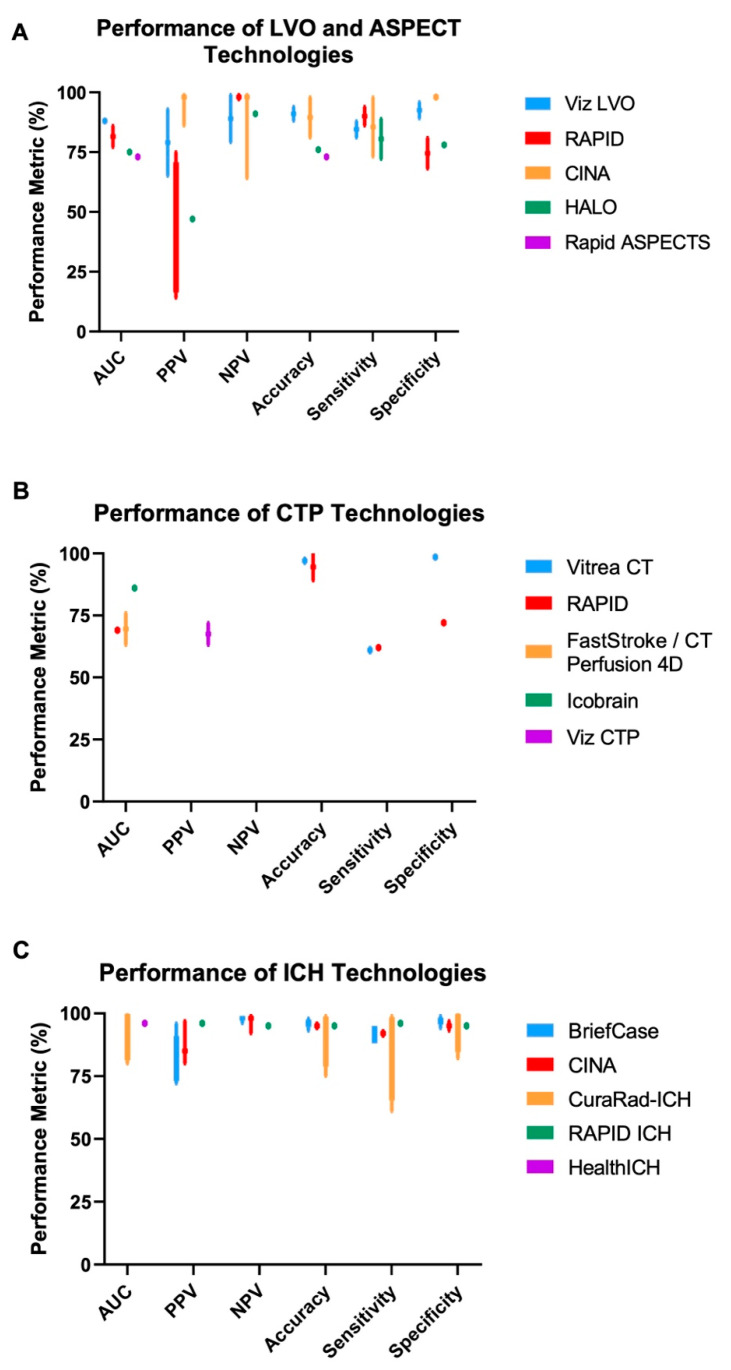
Reported technology performance from the literature. Various performance metrics (*x*-axis; using human performance as the gold standard) are reported for (**A**) LVO and ASPECT technologies, (**B**) CTP technologies, and (**C**) ICH technologies. Bars represent the minimum and maximum reported values for each metric. Data are depicted as a single point when only one value is reported in the literature. Not every technology has data for all performance metrics. Variation in performance was generally higher for LVO/ASPECT technologies compared to ICH technologies. More performance quantification is needed for CTP technologies. ASPECT: Alberta Stroke Program Early CT Score; AUC: Area Under the Receiver Operating Curve; CTP: Computed Tomography Perfusion; ICH: Intracranial Hemorrhage; LVO: Large Vessel Occlusion; NPV: Negative Predictive Value; PPV: Positive Predictive Value. Metrics for Rapid ASPECTS are related to calculating ASPECT scores, while other technologies in (**A**) are evaluated on LVO detection.

An important application of AI/ML is the automated detection of large vessel occlusions. Viz ContaCT, commercially known as Viz LVO, was the first FDA-approved AI/ML-enabled technology indicated for stroke and uses a convolutional neural network (CNN) as the underlying algorithm to detect LVOs from CT angiography (CTA). In data submitted to the FDA, Viz LVO displayed an area under the receiver operating curve (AUC) of 0.91 and reduced time from scan reading to specialist notification from 58 to 7 min [20], indicating improvement of clinical workflow efficiency. Others found similar increases in efficiency when using Viz LVO, reporting decreased transfer and stroke team notification times [21,22], as well as lengths of stay in the neurological ICU [22] (Table 2). Assessment of Viz LVO’s performance has shown negative predictive values (NPV) ranging from 79 to 99% and sensitivities between 81 and 88%, with relatively fast run times (~3 min) and consistent performance across different vascular structures [23,24] (Figure 2A). Notably, Viz LVO is an application within the broader Viz.ai platform, which includes tissue perfusion analysis on CTP and ICH identification on CT of the head.

RapidAI is a technology platform similar to Viz.ai. In addition to LVO identification on CTA (RAPID-CTA, RAPID-LVO), RapidAI includes software to analyze CT perfusion (RAPID-CTP) and MRI (RAPID-MRI) images for stroke triaging [36]. Though RAPID-LVO has a reported NPV range of 97–99% [26] and sensitivity ranging from 80–94%, there is a wide range of reported positive predictive values (PPV). Importantly, the PPV is 14% when identifying LVOs in the M2 segment of the MCA [26]. This is in contrast to Viz LVO’s reported lower bound PPV of 65%, which did not vary significantly across ICA, M1-MCA, and M2-MCA [24]. Variations in and relatively low PPVs highlight the use of these platforms as initial screening tools (given their high sensitivities and negative predictive values) that require subsequent expert confirmation to determine the presence of LVO (Table 2, Figure 2A). Use of both RAPID and Viz LVO has improved clinical workflows/outcomes (e.g., reducing CT to groin puncture times) with similar run times of ~3 min per scan [21,22,25].

Newer technologies for LVO identification include CINA-LVO [37] and HALO [38], which have shown promising performance in the few studies that have assessed their functionality. CINA has demonstrated relatively strong performance (PPV of 86–99%, NPV of 64–99%) across LVO anatomy [27,28]. The limited data for HALO reports an NPV of 91% and a PPV of 47%; however, performance varied based on the anatomical location of the LVO, with the lowest performance in M2 LVOs [29].

## 5. CT Head (CTH) Analysis (ASPECTS Score) in Acute Ischemic Stroke

Assessing the extent of irreversible ischemic damage to guide treatment decisions is equally important as identifying suspected LVOs. The Alberta Stroke Program Early CT Score (ASPECTS) is one widely used method for accomplishing this task. While diffusion-weighted MR imaging provides the most accurate information regarding acute infarction, CTH is more readily available in the acute setting. FDA-approved Rapid ASPECTS determines ASPECTS from CTs in patients with known MCA or ICA occlusions, but not for primary interpretation of CT images. In addition, the technology is only intended for use on GE Lightspeed VCT Scanners [39]. Overall, many have shown a strong correlation between ASPECTS determined manually by experts (e.g., neuroradiologists), which is currently the gold standard, and those calculated by Rapid ASPECTS [30,31,35] (Table 2). Some even report superior performance by Rapid ASPECTS in analyzing imaging obtained soon after symptom onset [32,34]. Rapid ASPECTS’ individual impact on clinical efficiency and patient outcomes has not yet been studied. However, use of the broader RapidAI mobile app, which includes Rapid ASPECTS functionality, decreased door-to-groin puncture times and improved subsequent NIH stroke scale scores [33].

## 6. CT Perfusion (CTP) Analysis in Acute Ischemic Stroke

Another class of FDA-approved, AI/ML-enabled technologies for the management of stroke includes technologies that analyze CTP or MR perfusion images to assess the core and penumbra volumes and predict final infarct volumes. CTP can demonstrate ischemic tissue, which consists of non-salvageable tissue and at-risk tissue that could be rescued with successful reperfusion. CTP analysis provides specific parameters, including cerebral blood volume (CBV), cerebral blood flow (CBF), and mean transit time (MTT). Rapid-CTP is a comprehensively studied tool for CTP analysis within the broader RAPID platform and performs well in estimating final infarct volumes, with high accuracy and relatively strong correlations to the gold standard (e.g., human estimates of volumes) [40,41,42,43,44,45] (Table 3). Vitrea CT Brain Perfusion was approved by the FDA in November 2018 to quantify cerebral blood flow and predict final infarct volumes [46]. Many groups have found Vitrea outperforms Rapid-CTP with respect to final infarct volume predictions [47,48,49], with the gold standard determined by human interpretation of DWI/FLAIR imaging (Table 3; Figure 2B). FastStroke/CT Perfusion 4D is a similar technology that not only predicts ischemic core volume but also assesses the quantity and quality of collateral perfusion [50,51]. Similar to Vitrea CT, FastStroke/CT Perfusion 4D performed comparably to Rapid-CTP (intraclass correlation coefficient of 0.95) [52], and its additional capability to assess collateral circulation improved accuracy in predicting good outcomes [53]. Icobrain CTP uses a CNN to estimate penumbra volumes and cerebral blood flow, both of which have strong correlations to expert assessments by radiologists [54,55] (Table 3). Viz CTP is a similar software that performed well in predicting final infarct volume (r = ~0.6) [56]. While the above software solutions are well-characterized, there are no studies demonstrating improved time-to-reperfusion. Solutions such as Augmented Vascular Analysis [57] and Neuro.AI Algorithm [58] are yet to be independently assessed in the literature (Table 3).

## 7. Intracranial Hemorrhage (ICH) Identification

Technologies indicated for the detection of ICH generally performed better than those indicated for LVO detection (Table 4). BriefCase was the first FDA-approved, AI/ML-enabled technology for the identification of ICH from non-contrast head CT [62]. BriefCase’s CNN-based algorithm [63] has shown strong performance by reducing outpatient scan interpretation delays by 90% (604 min reduction) and inpatient delays by 10% (38 min reduction) [64]. Cases flagged by BriefCase as suspicious for ICH had an average turnaround time of 73 min, versus 132 min for non-flagged cases [65]. Recent studies assessing BriefCase have reported NPVs of 96–99% and PPVs of 72–96% [64,66,67] (Figure 2C). A main driver of false negatives was ICH anatomy (e.g., under the calvaria), while false positives were driven by tumors and calcifications [68,69].

CINA-ICH has similar reported performance in ICH detection compared to BriefCase (Figure 2C). NPVs ranged from 92–99%, PPVs from 80–97%, and the algorithm had a sensitivity of 72% when identifying relatively small-volume bleeds (volume less than 5 mL) [27,70]. CINA has additional subclassification functionality (e.g., differentiating between subarachnoid and intraventricular hemorrhage) with a sensitivity of at least 90% [27]. CuraRad-ICH, on the other hand, had subclassification sensitivities between 61 and 99% [71,72], though the software was studied on a larger sample of scans and has specificities roughly comparable to those of CINA.

Rapid-ICH [73], with PPV, NPV, accuracy, sensitivity, and specificity of at least 95% [74], and HealthICH [75], with an AUC of 0.96 [76], are two other technologies indicated for ICH detection. Some FDA-approved technologies for ICH detection have yet to be studied independently in the literature. These include Accipiolx [77], DeepCT [78], NinesAI [79], qER [80], and Viz ICH [81].

**Table 4:** Ten technologies indicated to diagnose ICH. The majority of studies were retrospective, and algorithm performance met or exceeded human performance in binary and multiclass classification. ICH location (e.g., under the calvaria) and anatomical variations (e.g., calcification of the falx) reduced algorithm performance. Human performance generally continues to be the gold standard for evaluating these algorithms. CPH: Cerebral Parenchymal Hemorrhage; EDH: Extradural hemorrhage; ICA: Internal Carotid Artery; IVH: Intraventricular Hemorrhage; MCA: Middle Cerebral Artery; SAH: Subarachnoid Hemorrhage. * Indicates metric was extrapolated from available data.

Device	Author, Year	Level of Evidence	Dataset Characteristics	Sample Size (Scans)	AUC	PPV	NPV	Accuracy	Sensitivity	Specificity	Other Metrics/Comments
BriefCase	Ojeda et al., 2019 [63]	Retrospective	Proprietary, Multicenter	7112	-	96%	98%	98%	95%	99%	BriefCase uses a CNN to analyze non-contrast CTs to detect and triage ICH.
	Wismüller et al., 2020 [65]	Randomized Clinical Trial	Proprietary, Single Center	620	-	-	-	96%	95%	97%	Turn-around times for cases flagged by BriefCase (73 min) were significantly lower than those for non-flagged cases (132 min).
	Ginat et al., 2020 [66]	Prospective	Proprietary, Single Center	2011	-	74%	98%	93%	89%	94%	Accuracy was significantly higher for emergency (96.5%) vs. inpatient (89.4%) cases. False positives had various causes, including: (1) artifacts, (2) thick dura, (3) intra-arterial clot, (4) calcifications, and (5) tumors.
	Rao et al., 2021 [69]	Retrospective	Proprietary, Single Center	5585	-	-	-	-	-	-	When applied to scans that radiologists reported as negative for ICH, BriefCase found 28 scans with ICH, of which 16 truly did. Subset analysis showed a false positive rate of 32%.
	Ginat et al., 2021 [64]	Retrospective	Proprietary, Single Center	8723	-	86%	96%	-	88%	96%	Scan view delay for cases flagged by the software decreased by 37 min for inpatients and 604 min for outpatients. In the ER, time reduction was most prominent during the 9 p.m. to 3 a.m. and 10 a.m. to 12 p.m. periods, and especially during the weekend.
	Voter et al., 2021 [67]	Retrospective	Proprietary, Single Center	3605	-	81%	99%	96% *	92%	98%	Neuroradiologists and the software agreed 97% of the time. Prior neurosurgery decreased model performance.
	Kundisch et al., 2021 [68]	Retrospective	Proprietary, Multicenter	4946	-	72% *	99% *	97% *	88% *	98% *	Software detected 29 additional ICHs (0.59%) in the cohort. False negative rate was 12.4% compared to the radiologist rate of 10.9%. Anatomical variations (e.g., calcifications) were difficult for the algorithm to analyze.
CINA	McLouth et al., 2021 [27]	Retrospective	Proprietary, Multicenter	814	-	80–97%	92–99%	96%	91%	97%	True positive rates (sensitivity) for ICH subclassification were >90%. ICH < 5 mL had a sensitivity of 72%.
	Rava et al., 2021 [70]	Retrospective	Proprietary, Single Center	302	-	85%	98%	94%	93%	93%	95% of ICH volumes were correctly triaged. 88% of non-ICH cases were correctly classified as ICH negative.
CuraRad-ICH	Ye et al., 2019 [71]	Retrospective	Proprietary, Multicenter	2836	0.8–1.0	-	-	75–99%	61–99%	82–99%	Algorithm was evaluated for binary classification (ICH vs. no ICH) and multi-type classification (CPH, SAH, EDH, SDH, IVH).
	Guo et al., 2020 [72]	Retrospective	Proprietary, Multicenter	1176	0.85–0.99	-	-	90–98%	78–97%	92–100%	Algorithm was evaluated for binary classification (ICH vs. no ICH) and multi-type classification (CPH, SAH, EDH, SDH, IVH).
Rapid ICH	Heit et al., 2021 [74]	Retrospective	Proprietary, Multicenter	308	-	96%	95%	95% *	96%	95%	
HealthICH	Bar et al., 2018 [76]	Retrospective	Proprietary, Multicenter	1426	0.96	-	-	-	-	-	
Accipiolx											
DeepCT											
NinesAI											
QER											
Viz ICH											

## 8. Rehabilitation

Tools for post-stroke rehabilitation require further development, especially given the poor natural recovery that is often seen with stroke [62]. There is a need for technologies that can extend the therapeutic window for patients and/or enable neurological recovery. Here, we describe two FDA-approved technologies that can enhance post-stroke recovery.

An Israeli-based company, BrainQ, is developing a non-invasive brain-computer interface (BCI) device that leverages extremely low frequency and low intensity electromagnetic fields (ELF-EMF) to promote post-stroke recovery [82,83]. After a stroke, patients often have abnormal neural oscillatory patterns, and exposure to tuned EMFs can influence these oscillations [84], thereby promoting periods of neuroplasticity [85,86]. BrainQ’s technology uses ML to extract motor-related spectral features from electrophysiology measurements (EEG, MEG/EMG) [87] and then translates these into a specific ELF-EMF treatment for patients [88].

BrainQ received FDA breakthrough status in February 2021 based on results from a pilot trial of 25 patients with a history of sub-acute ischemic stroke. Patients who received 40 min of ELF-EMF treatment 5 days a week for 8 weeks had superior recovery compared to the sham group as assessed by multiple metrics (e.g., NIH stroke score) and did not report any adverse events [89]. BrainQ has planned a double-blind national clinical trial across up to 20 inpatient rehabilitation facilities in the United States [90]. A previous BrainQ clinical trial was terminated due to the COVID-19 pandemic [91].

IpsiHand Upper Extremity Rehabilitation System (IpsiHand), granted breakthrough status by the FDA in April 2021, is the first FDA-approved device to use BCI technology to facilitate motor rehabilitation in patients who are more than 6 months post-stroke. The device uses an EEG electrode headset to translate neural activity of movement intent from the uninjured brain hemisphere into physical movements of a robotic exoskeleton worn around the impaired hand, wrist, and forearm [92]. A study of ten chronic hemiparetic stroke survivors with upper-limb impairment showed significant improvement in arm functionality after 12 weeks of IpsiHand therapy, with only minor side effects (e.g., skin redness) [93]. A randomized clinical trial is needed to assess whether use of IpsiHand alone proves more beneficial for upper extremity function versus traditional physical therapy. IpsiHand has the potential to enhance functional recovery with convenient, in-home post-stroke rehabilitation.

## 9. Discussion

In total, the FDA has approved 22 unique AI/ML-enabled technologies to assist clinicians with the diagnosis or management of stroke or ICH. The 20 technologies indicated for assistance with diagnosis can save valuable time by triaging potentially troubling scans and reducing the need for labor-intensive and time-consuming tasks such as segmentation. These technologies can ultimately reduce delays for patients to receive life-saving interventions. Adoption of these technologies has been strong, with RAPID in use at 1800 and Viz.ai in 900 hospitals [94,95]. In addition, some of the technologies described here can co-function within a broader technology suite to facilitate care coordination. For example, technologies developed by the same company (e.g., Viz.ai, RapidAI) are hosted within an interconnected system that includes mobile alerts, capabilities for remote CT/MRI viewing, and HIPAA-compliant provider-to-provider communication. This simplification of care coordination works synergistically with AI/ML capabilities to achieve the results these technologies have produced. In the future, more of these technologies will need to be housed within similar, integrated clinical systems. Such technology-based care coordination and workflow simplification solutions have improved outcomes in non-stroke medical emergency settings [96]. The two devices indicated for post-stroke rehabilitation are leveraging AI/ML to create new forms of therapies that are opening the possibility for patients to achieve significant recovery following serious neurological injury. With time, the safety and capabilities of these devices will only improve, further enabling clinicians to facilitate favorable patient outcomes. In the future, there will be a need for head-to-head comparisons of technologies using the same clinical datasets to further help clinicians and health systems more definitively decide which to use.

Given the proprietary nature of the technologies discussed here, specific details about ML model design and algorithms are not publicly available. However, most models that underlie technologies indicated for stroke diagnosis leverage deep learning in the form of convolutional neural networks. The reasons for performance variation across these technologies are multifactorial and likely due to a combination of model design and quality/quantity of testing data. Model design in ML development involves determining model structure (e.g., number of layers in a neural network) and hyperparameters (e.g., learning rate for optimization algorithms) and still relies on trial and error [97,98,99,100]. When testing different model designs, developers must also keep end-user performance and experience in mind, as these technologies must work across multiple imaging platforms and user devices. Differences in the quality and quantity of training data also likely contribute to differences in performance among the technologies discussed here. Currently, the “gold-standard” used to train and measure the success metrics of the models underlying these technologies is produced by radiologists and other clinicians. Therefore, any inherent human error in this data will be carried forward and learned by the algorithm. This is primarily an issue for algorithms that require segmentation for tasks such as CT perfusion analysis and ICH detection. Classification algorithms (e.g., triaging CT scans for stroke) are less impacted by this human error, but their performance is influenced by the specific training methods (e.g., cross-validation, dropout regularization) that were employed during algorithm development. Finally, the size and variety of training data play a large role in algorithm performance. Training a model with a large number of unique data points is important to minimize overfitting and thereby maximize performance. The majority of the publications analyzed here used proprietary, single-center datasets to evaluate technologies (Table 2, Table 3 and Table 4) and there is a need for larger, multi-center, international research in order to more comprehensively test these technologies across a variety of clinical scenarios and patient populations.

There is great promise for AI/ML-enabled technologies in stroke diagnosis and management. The automated process can minimize intra-/inter-rater variability and provide support for less experienced or non-specialized physicians. As such, integration of these software solutions into patient care can improve the speed and accuracy of diagnosis. However, there are still issues to be addressed. The first is improving algorithm performance. Though many studies have shown that these AI/ML algorithms can perform comparably to or even outperform neuroradiologists, the technologies can fall short in certain instances. The anatomical location of the disease or patient-to-patient anatomical variation is a common cause of impaired algorithm performance. Difficulties with LVO identification were seen with anatomical variations such as early unilateral MCA or petrous ICA [26]. Additionally, LVO identification is less reliable for the posterior circulation, and algorithms encounter difficulty determining distal versus proximal occlusions. Similarly, AI-enabled software has had difficulty identifying ICH immediately under the calvaria [68], likely driven by a combination of beam-hardening and partial volume artifacts in CT imaging [101]. Anatomical variation, such as calcification of the falx cerebri, can also make ICH identification difficult [69]. Continued training of algorithms in patients with anatomical variations or additional intracranial abnormalities will be needed to improve performance. Fortunately, training functionalities can be developed that enable clinicians to inform the algorithm when errors are made. As discussed above, there is still a lack of robust, head-to-head comparisons between these various technologies, making it difficult to clearly identify one as “superior” to others. The technologies discussed here can safely be used in clinical practice to augment the expertise of radiologists, neurologists, and neurosurgeons, keeping in mind the performance data and shortcomings presented here.

The second issue to address with the use of these technologies involves the use of black-box AI/ML algorithms. Neural networks, and “deep” neural networks in particular, can tend to be “black box” algorithms in that they arrive at conclusions and outputs without readily providing information regarding their analytical process. As neural networks tend to underlie many AI/ML-enabled tools in healthcare today, some have argued that these black-box algorithms must have increased transparency before being used in high-stakes healthcare settings. There have been efforts to make AI/ML algorithms more interpretable to the end user [102,103], with current “explainability methods” satisfying desires for transparency to various extents [104]. Two common methods include saliency maps, which visually highlight key features that an algorithm used to make a decision, and feature relevance, which is a list of key quantitative or qualitative features that an algorithm used in decision-making [105]. The field should also focus on rigorous internal and external validation of algorithms and compliance with a common set of machine learning best practices that support a level of standardization within the field. The FDA’s plan to develop new protocols for assessing AI/ML-enabled technologies also addresses these concerns [6]. The role of intellectual property is a balancing concern that must be acknowledged. Though it would be beneficial for end users to understand how exactly a decision was made to, for example, not triage a scan for possible LVO, technology developers may worry that publicly releasing too much information could potentially compromise security of intellectual property. Still, there must be a common ground on which adequate information is given to users or regulators about how, for example, a piece of software makes decisions when analyzing radiology images.

The last issue that must be addressed involves reimbursement for hospitals that utilize these technologies. In 2018, the Centers for Medicare and Medicaid Services (CMS) approved payment for use of Viz LVO through a “New Technology Add-on Payment”, marking the first time CMS reimbursed AI/ML-enabled software through this mechanism [106]. RapidAI also received this designation from CMS [107], marking important administrative milestones to increase the use of these technologies in direct patient care. There is little public data available regarding the pricing of these technologies, and specific pricing structures likely vary between contracts across different hospitals. In the future, health systems must be aware of the reimbursement they receive when using these novel technologies when negotiating licensing contracts. Additional potential costs to a health system that must also be considered include increased expenditure on computing power and physical or cloud storage.

Overall, the studies analyzed here show that AI/ML-enabled technologies for stroke diagnosis are performing equally to or even exceeding human performance on certain metrics. Generally, these technologies have higher sensitivities and NPVs compared to specificities and PPVs, further highlighting their role in triaging and assisting clinicians rather than making definitive clinical decisions. In the coming years, as these technologies are exposed to more cases and increased clinical data, their performance should only improve, but as stated above, there is still a need for larger, multi-center trials to continue monitoring and comparing technology performance. There is a common theme in the studies assessed here: the use of these technologies can improve clinical workflows. However, few studies further correlated increased efficiency with quantifiable improvements in patient outcomes. Some studies report improvements in, for example, the NIH stroke scale, but there is still perhaps a need for long-term follow-up to assess if the workflow efficiency facilitated by these technologies truly translates to better long-term clinical outcomes. Similarly, there are inconsistencies across studies regarding the timing of imaging analysis, as only a few assessed the differential impact of using AI/ML-enabled technologies in the acute (e.g., 0–6 h) versus the sub-acute (e.g., 6–24 h) timeframes. This is particularly important when analyzing zones of ischemia and the viability of tissue post-stroke. In the same vein, there must be more consistency in reporting the time technologies take to evaluate imaging. As the complexity of medical imaging increases, the algorithms underlying these technologies must be refined to efficiently process scans at a speed that is at least equivalent to, if not faster than, human performance.

To facilitate the approval and clinical utilization of emerging AI/ML-enabled technologies, the FDA has created protocols to better assist researchers in developing these technologies and navigating the FDA approval process [6]. These protocols outlined good machine learning practices for researchers to follow, created guidelines for algorithm transparency, and established more robust guidelines for real-world data collection. As the field of AI/ML rapidly advances, these protocols must also undergo rapid revision to remain relevant. This includes new guidelines related to data security, bias in data sources, and post-approval monitoring. In the future, when more technologies are developed not only for stroke diagnosis but also for stroke prediction in high-risk populations and chronic stroke management, robust guidelines will be essential as these technologies are used in larger patient populations.

Our study has a few limitations. First, though the majority of papers analyzed here were identified in PubMed, some papers were only discovered after searching company websites. This may have introduced selection bias to our literature search, though the minority of papers were found by searching company websites. Second, our study focuses on FDA-approved solutions, but there are many emerging technologies in the research pipeline that are not discussed here. Third, given the proprietary nature of the technologies described here, we are unable to specifically comment on the strengths and weaknesses of the design of the algorithms that underlie these technologies.

## 10. Conclusions

FDA-approved AI/ML-enabled technologies for stroke diagnosis and management have proven to be powerful tools in improving the efficiency and accuracy of patient care decisions by physicians. Many of these technologies use convolutional neural networks as their underlying algorithm and have approached or even exceeded the gold standard of human performance when tested using real-world data. Future work is needed to further refine the performance of these technologies in, for example, patients with aberrant anatomy and to compare technologies in a head-to-head manner with large, multi-center studies.

## Figures and Tables

**Table 1:** List of 22 unique AI/ML-enabled, FDA-approved technologies indicated for diagnosis and/or management of stroke. Twenty technologies are indicated for stroke diagnosis and two for post-stroke rehabilitation, with approval dates ranging from February 2018 to April 2021. Eighteen companies developed these technologies, with 11/18 (61%) headquartered outside the United States. Generally, the technologies indicated for stroke diagnosis utilize convolutional neural networks as their underlying algorithm. CTA: Computed Tomography Angiography; CTP: Computed Tomography Perfusion; ICH: Intracranial Hemorrhage; LVO: Large Vessel Occlusion. * RAPID is indicated for both LVO and CTP analysis. ** CINA is indicated for both LVO and ICH analyses. 510(k) refers to a premarket submission made to the FDA demonstrating the proposed device to be as safe and effective as a legally marketed device. 513(f)(2) (De Novo) classification describes devices that are considered for Class I (low-to-moderate risk) or II (moderate-to-high risk) categorization, either after receiving a “not substantially equivalent” determination post-510(k) submission or in cases where there is no legally marketed device to determine substantial equivalence. A breakthrough status designation aims to expedite the development, assessment, and review of a medical device to provide patients with timely access to such a device while maintaining FDA statutory standards in the approval process.

Device	Company Name	Headquarters	FDA Approval Number	Type of Approval	Indication	Date of Approval
ContaCT (Viz LVO)	Viz.ai	San Francisco, CA, USA	DEN170073	513(f)(2)(De Novo)	Analyze acute CTA to identify LVO	February 2018
Viz CTP	Viz.ai	San Francisco, CA, USA	K180161	510(k)	Analyze brain tissue perfusion parameters on CTP	April 2018
BriefCase	Aidoc Medical	Israel	K180647	510(k)	ICH detection in non-contrast CT	August 2018
Accipiolx	MaxQ Al	Israel	K182177	510(k)	ICH detection in non-contrast CT	October 2018
Vitrea CT Brain Perfusion	Vital Images	Minnetonka, MN, USA	K181247	510(k)	Visualize apparent blood perfusion in brain tissue affected by acute stroke on CT	November 2018
RAPID *	iSchemaView	Golden, CO, USA	K182130	510(k)	Identification of CTP, CTA, and MRI images consistent with stroke	December 2018
HealthICH	Zebra Medical Vision (now Nanox AI)	Israel	K190424	510(k)	Aid clinical assessment of non-contrast head CT with features suggestive of ICH	June 2019
DeepCT	Deep01 Limited	Taiwan	K182875	510(k)	ICH detection in non-contrast CT	July 2019
Icobrain-CTP	Icometrix	Belgium	K192962	510(k)	Image analysis of brain CT perfusion scans	February 2020
Rapid ICH	iSchemaView	Golden, CO, USA	K193087	510(k)	ICH detection in non-contrast CT	March 2020
CuraRad-ICH	CuraCloud	Seattle, WA, USA	K192167	510(k)	ICH detection in non-contrast CT	April 2020
NinesAI	Nines	Palo Alto, VA, USA	K193351	510(k)	ICH detection in non-contrast CT	April 2020
CINA **	AVICENNA.AI	France	K200855	510(k)	ICH in head CT and LVO in head CT angiography	June 2020
Rapid ASPECTS	iSchemaView	Golden, CO, USA	K200760	510(k)	ASPECT scoring in patients with known MCA or ICA occlusions	June 2020
QER	Qure.Ai Technologies	India	K200921	510(k)	ICH detection in non-contrast CT	June 2020
Augmented Vascular Analysis	See-Mode Technologies	Singapore	K201369	510(k)	Predict stroke risk from vascular ultrasound	September 2020
HALO	NICo-Lab B.V.	Amsterdam	K200873	510(k)	LVO identification in anterior circulation (ICA, M1 or M2) from CT angiogram	November 2020
FastStroke, CT Perfusion 4D	GE Medical Systems SCS	France	K193289	510(k)	CT Perfusion 4D: Perfusion abnormalities from contrast CTFastStroke: Stroke detection from CT (e.g., non-constast, angiogram)	November 2020
Neuro.Al Algorithm	TeraRecon	Durham, NC, USA	K200750	510(k)	Detect changes in brain perfusion from CT or MRI	November 2020
BrainQ	BrainQ	Israel	-	Breakthrough status	Reduce disability post-stroke	February 2021
Viz ICH	Viz.ai	San Francisco, CA, USA	K210209	510(k)	Analyze acute non-contrast CT of brain, notify specialist of suspected ICH	March 2021
IpsiHand Upper Extremity Rehabilitation System	Neurolutions	Santa Cruz, CA, USA	-	Breakthrough status	Post-stroke rehabilitation	April 2021

**Table 2:** Five technologies are indicated to diagnose LVO and calculate the Alberta Stroke Program Early CT Score (ASPECTS). The majority of studies assessing these technologies were retrospective. Algorithm performance either met or exceeded human performance, and implementation of the solutions has improved clinical workflows and patient outcomes. Difficulties with LVO identification were occasionally seen with vessel anatomical variation. Human performance generally continues to be the gold standard for evaluating these algorithms. ICA: Internal Carotid Artery; MCA: Middle Cerebral Artery. * Indicates metric was extrapolated from available data.

Device	Author, Year	Level of Evidence	Dataset Characteristics	Sample Size (Scans)	AUC	PPV	NPV	Accuracy	Sensitivity	Specificity	Other Metrics/Comments
Viz LVO	Hassan et al., 2020 [22]	Prospective	Proprietary, Single Center	43	-	-	-	-	-	-	Viz LVO reduced median CTA time at primary center to door-in at comprehensive center by an average of 22.5 min. Neuro-ICU stays were reduced by 2.5 days.
	Yahav-Dovrat et al., 2021 [23]	Prospective	Proprietary, Single Center	1167	-	65%	99%	94%	81%	96%	-
	Morey et al., 2021 [21]	Retrospective	Proprietary, Single Center	55	-	-	-	-	-	-	Viz LVO reduced median door-to-neurovascular team notification time from 40 to 25 min.
	Rodrigues et al., 2021 [24]	Retrospective	Proprietary, Single Center	610	0.88	93%	79%	88%	88%	89%	Algorithm had similar performance across ICA-T, MCA-M1, and MCA-M2 occlusions. Mean run time was ~3 min.
RAPID (LVO)	Adhya et al., 2021 [25]	Retrospective	Proprietary, Multicenter	310	-	23–75%	-	-	80%	-	CT to groin puncture time was lower after implementation of RAPID (93 min vs. 68 min).
	Amukotuwa et al., 2019 [26]	Retrospective	Proprietary, Single Center	477	0.77–0.86	14–58%	97–99%	-	86–94%	68–81%	Median scan analysis time was roughly 160 s.
CINA	McLouth et al., 2021 [27]	Retrospective	Proprietary, Multicenter	378	-	86–98%	98–99%	98%	98%	98%	In the detection of LVO subtypes (i.e., at distal internal carotid artery, middle cerebral artery M1 segment, proximal middle cerebral artery M2 segment, distal middle cerebral artery M2 segment), the CINA algorithm demonstrated an accuracy of 97%, sensitivity of 94.3%, and specificity of 97.4%.
	Rava et al., 2021 [28]	Retrospective	Proprietary, Single Center	303	-	99%	64%	81%	73%	98%	Scan processing time was ~70 s. The algorithm identified ICA, M1 MCA, and M2 MCA occlusions.
HALO	Luijten et al., 2021 [29]	Prospective	MR CLEAN registry & PRESTO study, Multicenter	1756	0.75	47%	91%	76% *	72-89%	78%	Performance varied considerably based on location of occlusion.
Rapid ASPECTS	Lasocha et al., 2020 [30]	Retrospective	Proprietary, Single Center	100	-	-	-	-	-	-	Exact ASPECT score agreement between RAPID and manual methods was poor, but crossing of threshold for reperfusion therapy was characterized by an 80% match.
	Hoelter et al., 2020 [31]	Retrospective	Proprietary, Single Center	131	0.73	-	-	-	-	-	Correlation between ASPECT scores of experts and RAPID was high (r = 0.78)
	Maegerlein et al., 2019 [32]	Retrospective	Proprietary, Single Center	100	-	-	-	-	-	-	In acute stroke of the middle cerebral artery, RAPID-calculated ASPECT score had better agreement with predefined consensus scores than neuroradiologists overall (κ = 0.9 vs. κ = 0.57, respectively), and particularly in the time interval of 1 to 4 h between symptom onset and imaging.
	Al-Kawaz et al., 2021 [33]	Retrospective	Proprietary, Single Center	64	-	-	-	-	-	-	Use of the RAPID mobile app (which includes Rapid ASPECTS functionality) decreased door to groin puncture times by 33 min compared to patients treated pre-app and improved scores on National Institutes of Health Stroke Scale 24 h after procedure (12.1 vs. 8.0) and at discharge (11.8 vs. 7.8)
	Albers et al., 2019 [34]	Retrospective	GAMES-RP trial, Multicenter	65	-	-	-	73%	-	-	RAPID ASPECTS was more accurate than clinicians (73% vs. 56%) in identifying early ischemia on DWI.
	Mansour et al., 2020 [35]	Retrospective	Proprietary, Single Center	122	-	-	-	-	-	-	Automated ASPECT score by the algorithm performed equally to scoring by neuroradiologists (κ = 0.8).

**Table 3:** Seven technologies are indicated to analyze CTP images. The majority of studies were retrospective. Algorithms are able to predict final infarct volume and/or assess the quality of collateral perfusion, and algorithm performance met or exceeded human performance in binary and multiclass classification. ICH location (e.g., under the calvaria) and anatomical variations (e.g., calcification of the falx) reduced algorithm performance. Human performance generally continues to be the gold standard for evaluating these algorithms. CBF: Cerebral Blood Flow; CBV: Cerebral Blood Volume; MTT: Mean Transit Time; SVD: Singular Value Decomposition.

Device	Author, Year	Level of Evidence	Dataset Characteristics	Sample Size (Scans)	AUC	PPV	NPV	Accuracy	Sensitivity	Specificity	Other Metrics/Comments
Vitrea CT Brain Perfusion	Rava et al., 2020 [47]	Retrospective	Proprietary, Single Center	105	-	-	-	-	-	-	In estimating infarct volume, Spearman correlation coefficient between Vitrea and DWI/FLAIR ranged from 0.71 to 0.77. Vitrea outperformed RAPID.
	Rava et al., 2020 [48]	Retrospective	Proprietary, Single Center	107	-	-	-	-	-	-	In estimating infarct volume, Spearman correlation coefficient between different algorithms within Vitrea (i.e., Bayesian and Singular Value Decomposition) and FLAIR MRI was 0.98 vs. 0.76-0.87 between RAPID and FLAIR MRI.
	Rava et al., 2021 [49]	Retrospective	Proprietary, Single Center	63	-	63–72%	-	-	-	-	-
	Rava et al., 2021 [59]	Retrospective	Proprietary, Single Center	108	-	-	-	96–98%	60–62%	98–99%	Vitrea overestimated infarct volume, but provided the most accurate penumbra assessment for patients treated conservatively.
	Ichikawa et al., 2021 [60]	Retrospective	Proprietary, Single Center	36	-	-	-	-	-	-	Vitrea’s Bayesian algorithm had better delineation of abnormal perfusion areas and estimation of infarct volume compared to the SVD implementation.
RAPID (CTP)	Hokkinen et al., 2021 [40]	Retrospective	Proprietary, Single Center	89	-	-	-	-	-	-	In patients presenting 6 to 24 hours from onset of symptoms, CTP-RAPID’s estimate of infarct volume correlated with follow-up imaging (r = 0.82). Correlation decreased (r = 0.58) in patients presenting 0 to 6 hours after symptom onset.
	Wouters et al., 2021 [42]	Randomized Controlled Trial	MR CLEAN trial & CRISP study, Multicenter	127	-	-	-	-	-	-	A new deep learning CNN model outperformed RAPID in predicting final infarct volume.
	Potreck et al., 2021 [43]	Retrospective	Simulation	53	-	-	-	-	-	-	Head motion during CT perfusion acquisition can impact infarct core estimates.
	Bouslama et al., 2021 [44]	Retrospective	Proprietary, Single Center	479	-	-	-	-	-	-	RAPID had moderate correlation with final infarct volumes (r = 0.42–0.44).
	Siegler et al., 2020 [61]	Retrospective	Multi-site registry, Multicenter	410	0.69	-	-	-	62%	72%	Stroke mimics can show abnormalities on RAPID CT analysis.
	Kim et al., 2019 [45]	Prospective	Proprietary, Single Center	296	-	-	-	89–100%	-	-	Interclass correlation between RAPID and manual measurements of infarct volume were 0.98, with RAPID underestimating volumes by ~2 mL on average.
FastStroke/CT Perfusion 4D	Verdolotti et al., 2020 [51]	Retrospective	Proprietary, Single Center	86	-	-	-	-	-	-	Algorithm is comparable in efficacy to the status quo in evaluating collateral circulation, but has simpler workflows and faster turnaround times, making use easier for radiologists.
	Ospel et al., 2021 [53]	Prospective	PRove-IT cohort study, Multicenter	285	0.63–0.76	-	-	-	-	-	Time-variant multiphase CTA (mCTA) maps produced by the software improved prediction of good outcomes and performed comparably to conventional mCTA in predicting infarct volume.
	Liu et al., 2021 [52]	Retrospective	Proprietary, Single Center	82	-	-	-	-	-	-	CT Perfusion 4D had ICC of 0.95 compared to RAPID in predicting core volumes. The algorithm also performed well for volumes ≤ 70 mL
Icobrain-CTP	de la Rosa et al., 2021 [54]	Retrospective	Public ISLES18 stroke database	156	-	-	-	-	-	-	Icobrain uses a CNN that does not need user input in the form of thresholding to assess perfusion. Estimations of penumbra volume using CBF, CBV, and MTT had strong correlation with assessments by radiologists.
	de la Rosa et al., 2021 [55]	Retrospective	Public ISLES18 stroke database	156	0.86	-	-	-	-	-	Icobrain performed comparably to expert estimates of cerebral blood flow based on 4D CTP scans.
Viz CTP	Pisani et al., 2021 [56]	Prospective	Proprietary database otherwise unspecified	242	-	-	-	-	-	-	Viz CTP performed well in predicting final infarct volume (r = 0.601).
Augmented Vascular Analysis											
Neuro.Al Algorithm											

## Data Availability

The data used in this study are publicly available to researchers through the U.S. National Library of Medicine. Additional inquiries are welcome to the corresponding author.
